# Pheochromocytoma presenting with arterial and intracardiac thrombus in a 47-year-old woman: a case report

**DOI:** 10.1186/1752-1947-5-310

**Published:** 2011-07-13

**Authors:** Runhua Hou, Ann M Leathersich, Brenda Temke Ruud

**Affiliations:** 1 Endocrine Unit, Department of Medicine, University of Rochester, Rochester, NY, 14642, USA; 2 Department of Pathology, Washington University School of Medicine, St Louis, MO, 63110, USA

## Abstract

**Introduction:**

Pheochromocytoma is a rare cause of hypertension but it could have severe consequences if not recognized and treated appropriately. The association of pheochromocytoma and thrombosis is even rarer but significantly increases management complexity, morbidity and mortality. To the best of our knowledge, this is the first report of a patient with pheochromocytoma presenting with left axillary arterial and intracardiac thrombus.

**Case presentation:**

A 47-year-old Caucasian woman with a past medical history of hypertension presented for medical attention with left arm numbness. Doppler ultrasound showed an obstructing thrombus in her left axillary artery. She had symptom resolution after stent placement in her left axillary artery. A subsequent echocardiogram demonstrated a large intracardiac mass and abdominal computed tomography revealed a 7 cm mass between her spleen and left kidney. Labile blood pressure was noted during admission and she had very high levels of plasma and 24-hour urine catecholamines and metanephrines tests. A (123)I- metaiodobenzylguanidine scan showed intense uptake in the left abdominal mass. After adequate alpha blockage with phenoxybenzamine, laparoscopic tumor resection was performed without complications. She had normal metanephrines and complete symptom resolution afterwards. The intracardiac mass also disappeared with anticoagulation. All other endocrine laboratory abnormalities returned to normal after surgery.

**Conclusion:**

Arterial and ventricular thrombosis occurring in patients with pheochromocytoma is rare. A multi-disciplinary approach is necessary in caring for this type of patient. Catecholamines likely contributed to the development of thrombosis in our patient. Early recognition of pheochromocytoma is the key to improving outcome.

## Introduction

Pheochromocytoma is a rare disease occurring in less than 0.2 percent of patients with hypertension [1,2]. The classic presentation includes episodic hypertension, headaches and palpitations. However, many patients may have atypical presentations which often delay diagnosis. Even with classical presentations, the diagnosis is often missed for a number of years unless the patient is evaluated in a center experienced in this disease. Pheochromocytoma can have devastating consequences if not recognized and treated appropriately. Thrombolic events have been reported rarely in patients with pheochromocytoma and the exact mechanism of thrombosis is unclear [3-8]. Here we report a patient with pheochromocytoma presenting with a left axillary arterial and intracardiac thrombus.

## Case presentation

A 47-year-old Caucasian woman with a past medical history of hypertension presented to a local hospital for acute onset of numbness of her left arm in May 2007. A left axillary arterial thrombus was identified on Doppler ultrasound. Subsequently, our patient underwent left axillary artery stent placement with complete symptom resolution. To identify the source of the thrombus an echocardiogram was performed, which revealed a large mobile mass adherent to the anterior apical region of her left ventricle. The left ventricle ejection fraction was normal at 60%. The intracardiac mass was thought to be a thrombus and anticoagulation was initiated to prevent further embolic events. Possible cancer-induced thrombosis was suspected and a computed tomography (CT) scan of her chest, abdomen and pelvis was obtained. This showed a 7 × 8 cm complex mass between the upper pole of her left kidney and her spleen as well as a 3 cm nodule in the right lower lobe of her lung. A total body positron emission tomography (PET) scan revealed increased uptake in the abdominal mass as well as the lung nodule, which raised the question of metastatic disease. Initially, renal cell carcinoma was considered the most likely diagnosis and surgery was scheduled to take place following dissolution of the intracardiac thrombus. However, while still hospitalized for anticoagulation therapy, our patient had multiple hypertensive episodes with a blood pressure as high as 223/139 mmHg. This prompted a 24-hour urine collection for catecholamine and metanephrine tests. Her 24-hour urine metanephrine and normetanephrine levels were significantly elevated at 18160 μg (normal range: 19-140 μg), and 7742 μg (normal range: 52-310 μg) respectively. Her 24-hour urine epinephrine level was 756 μg (normal range: 2-24 μg) and norepinephrine was 1161 μg (normal range: 15-199 μg). Her 24-hour urine vanillylmandelic acid (VMA) was also elevated at 46.6 μg (normal range: < 6 μg). She was then referred to our endocrine clinic for further management.

A review of her previous history revealed multiple "spells" occurring over the last five years. During these episodes, our patient experienced palpitations, a heavy pounding heart beat and a sensation of "my heart jumping out of my chest". They were accompanied by cold sweats and right-sided headaches. Occasionally, a left-sided burning sensation of the chest along with nausea and vomiting would accompany the headaches. The "spells" were sometimes associated with bending over or lying on her right side. Interestingly, these episodes occurred more often between 10 am and 11 am. The frequency of the spells had increased to almost daily over the two weeks prior to presentation. Normally her "spells" lasted only for a few seconds to a few minutes then disappeared completely. Her blood pressure has been normal until 2005 and she only started to take hydrochlorothiazide and lisinopril in 2006. During her "spell" at the outside hospital, it was noted that she was very hypertensive with a systolic blood pressure of more than 230 mmHg, but her blood pressure returned to baseline after the spell passed.

Her previous studies included a normal thyroid scan. Her non-specific symptoms were attributed to acid reflux. Her hypertension was treated with atenolol, lisinopril and hydrochlorothiazide prior to admission to the outside hospital. She does not have any other chronic illness. Her medication at presentation to our clinic included atenolol, hydrochlorothiazide, Plavix (clopidogrel), lisinopril, Prilosec (omeprazole), potassium chloride, and warfarin. She was on an oral contraceptive which was discontinued after her hospital admission. Her social history is unremarkable. Both of her parents have hypertension; otherwise there is no family history of multiple endocrine neoplasia type 2, Von Hippel-Lindau disease, neurofibromatosis, pheochromocytoma, thyroid cancers or any other endocrine tumors. Her review of systems was remarkable for fatigue, nasal congestion, and cough with greenish sputum production over the few weeks prior to presentation. Her physical examination was significant only for a blood pressure of 120/77 mmHg, a heart rate of 96 beats per minute, and a 2/6 systolic murmur over the precordial area.

Our patient had significantly elevated levels of plasma metanephrine at 14.5 nmol/L (normal range: < 0.49 nmol/L), and normetanephrine at 24.3 nmol/L (normal range: < 0.89 nmol/L). Similar to reports in the literature [9], she also had an increased white blood cell count (WBC) of 13.3 k/mm^3 ^(normal range: 3.8-9.8 k/mm^3^) and a platelet count of 629 k/mm^3 ^(normal range: 140-440 k/mm^3^). Her other endocrine studies showed a moderately elevated plasma renin level of 21.5 ng/ml/hr (normal range: 0.65-5.0 ng/ml/hr), abnormal fasting blood glucose (163 mg/dl) and hemoglobin-A1C (HbA1C) (6.8%). Her aldosterone and thyroid-stimulating hormone levels were within normal limits. She had no detectable cardiolipin antibodies, antinuclear antibody, factor V Leiden, or prothrombin gene mutation and her rheumatoid factor (RF) was within normal limits. Her homocysteine level was mildly elevated at 14 umol/L (normal range: < 10.4 umol/L). The DNA analysis for methylenetetrahydrofolate reductase (MTHFR) showed a heterozygous mutation for C677T and A1298C.

**Figure 1:**
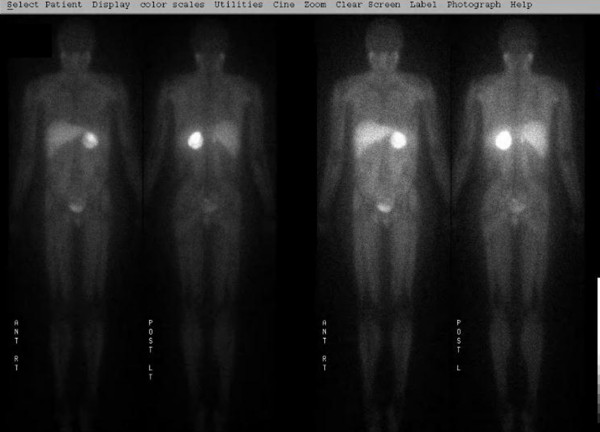
**^123^I-MIBG scan showing intensive uptake in the adrenal mass but no uptake in the lung**. A ^123^I-MIBG scan was obtained to determine whether the left adrenal mass and right lung mass are pheochromocytoma. Shown are the frontal and back views of the total body scan at 72 hours. Significantly increased uptake is seen in the left adrenal lesion. No uptake was found in the lung. Physiological uptake is seen in the salivary glands, heart and liver. ^123^I-MIBG is renally excreted and is visible in the bladder.

Based on the above data, our patient was diagnosed with pheochromocytoma and treatment with phenoxybenzamine at 10 mg once a day with gradual dose titration to 30 mg three times a day was initiated. The increased uptake of the right lower lobe of the lung on a PET scan raised the question of a metastatic lesion. Our patient experienced symptoms of cough, nasal congestion and green sputum production two to three weeks prior to presentation to the outside hospital. She likely had pneumonia at that time. Pneumonia may cause increased uptake on a PET scan as well. Nevertheless, a (123)I- metaiodobenzylguanidine (^123^I-MIBG) scan was indicated to further differentiate between metastasis and pneumonia. Her ^123^I-MIBG scan revealed intense uptake only in the left abdominal mass excluding the lung mass being metastatic (Figure 1).

**Figure 2:**
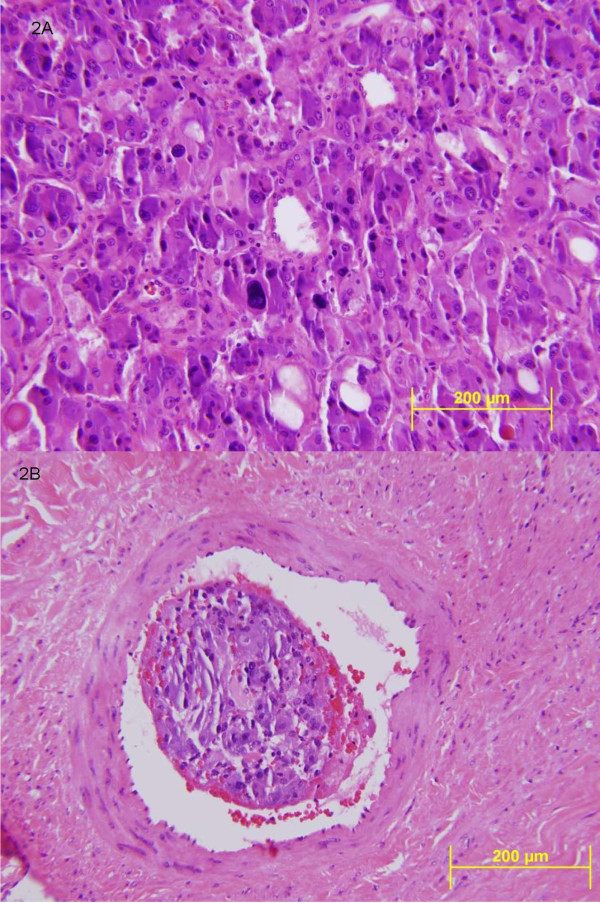
**Histological views of the resected adrenal tumor and its intravascular invasion**. **A**. High power (400 ×) view of the resected adrenal tumor. The resected adrenal pheochromocytoma shows chromaffin cells with a classic nested and trabecular architecture. Other characteristic morphologic features include nuclear enlargement and hyperchromasia with cytoplasm that is both oncocytic (pink and granular) in some cells and basophilic (blue) in others. **B.** Intra-vascular invasion of tumor (400 ×). Pheochromocytoma cells seen within a blood vessel. Vascular invasion is not a reliable indication of a malignant pheochromocytoma. Only metastatic disease to regional lymph nodes or distant sites (most commonly ribs, spine, liver and lung) will define this tumor as a malignant lesion.

Six weeks after initiating anticoagulation, the intracardiac mass was no longer present on repeat echocardiogram. The adrenal mass was removed by laparoscopy without complication. The surgical pathology report confirmed the diagnosis of a pheochromocytoma with the presence of vascular invasion by tumor (Figure 2). Six weeks post-operatively, repeat plasma metanephrine and normetanephrine levels were normal at < 0.2 nmol/L and 0.72 nmol/L and they remained normal at follow-up five months later (metanephrine < 0.2 nmol/L and normetanephrine 0.85 nmol/L). The other laboratory abnormalities such as HbA1C, fasting blood glucose, renin, platelet, and WBC all returned to normal. A few months after surgery, a follow-up chest X-ray showed no evidence of a lung mass. Our patient did not experience any more "spells."

## Discussion

In this report, we describe a patient presenting with left arm numbness, who was later discovered to have left ventricular thrombus, left axillary arterial thrombus and a large left adrenal pheochromocytoma. Her presentation is unusual in that she had a thrombolic event before the diagnosis of pheochromocytoma.

Cardiac thrombosis generally occurs as a result of decreased ventricular contraction in the setting of anterior or apical wall myocardial infarction. It could also happen in patients with normal ventricular contraction in the setting of a hypercoagulative state, such as in patients with cancer. It is often a result of inflammatory cytokines (tumor necrosis factor, interferon-γ), coagulation proteins (tissue factor and factor VIII), and pro-coagulants secreted by tumor cells [10]. It generally occurs late in the progression of carcinomas and is considered a poor prognostic sign.

Thrombosis occurring in patients with pheochromocytoma has been reported previously in only a few cases [3-8]. In one report, diffuse venous thromboses occurred in a patient with malignant pheochromocytoma, multiple metastasis and polycythemia [5]. In another case, central venous thrombosis occurred in conjunction with pheochromocytoma and diabetes insipidus [6]. Additionally, a left ilio-femoral venous thrombosis was reported in a patient with pheochromocytoma within the organ of Zuckerkandl [7]. Besides venous thrombosis, cardiac thrombosis has been reported in only three cases. One patient presented with shortness of breath, a left ventricular mass and later developed a left frontal lobe infarct [3]. During cardiac surgery to explore the intracardiac mass, significant blood pressure fluctuation was noted and surgery had to be aborted. This patient was later found to have a 10 cm intra-adrenal mass. In another case, a patient with medullary thyroid cancer and adrenal pheochromocytoma developed a left ventricular mass which was proven surgically to be a thrombus and, similar to our patient, there was no evidence of ventricular wall contraction abnormalities [4]. The most recently reported pheochromocytoma case described a patient with a large left ventricular thrombus and a 7 cm right adrenal mass [8]. Unfortunately, without prompt anticoagulation, the patient developed systemic embolization leading to kidney infarction and lower extremity infarction requiring bilateral below-the-knee amputation. In review of the above cases, definite underlying coagulation defects were rarely identified, whereas erythropoietin, pro-coagulant and serotonin secreted by the pheochromocytoma are postulated to be contributing factors. Interestingly, in some of the cases where surgery was possible, recurrence of thrombosis was not reported after resection of the pheochromocytoma. Therefore, it is likely that catecholamines and other hormones, cytokine or factors secreted by pheochromocytoma may play an important role in the pathogenesis of concurrent thrombosis and pheochromocytoma when a predisposing coagulation disorder is not identified.

The possibility of an intracardiac mass being pheochromocytoma was also entertained in our patient. Intracardiac pheochromocytoma is very rare. In a report of 32 cases, 19 cases were in the left atrium, seven in the inter-atrial septum and the remaining six on the anterior surface of the heart [11]. None of them were in the left ventricle. In another report, pheochromocytoma were found on the left atrial surface, the atrio-ventricular groove, the left or right atrial cavity, the aorto-pulmonary window and the aortic root [12]. A pheochromocytoma has not been reported in the left ventricle. The fact that the intracardiac mass in our patient resolved following anticoagulation and was negative on the ^123^I-MIBG scan supported the diagnosis of a thrombus rather than intracardiac pheochromocytoma.

The thrombosis in our patient may be multi-factorial, but pheochromocytoma probably played an important role. It has been reported that platelet aggregation is increased in patients with a pheochromocytoma [13,14]. This may predispose a patient to form thrombi in low-flow areas and increase acute coronary events. However, moderately elevated platelet counts (less than 1000 k/mm^3^) are often not considered a significant risk factor. It is unknown whether moderately elevated platelet counts combined with a significantly elevated catecholamine level may significantly increase the risk of thrombosis. In this case, the platelet elevation could be reactive as it was only transiently elevated and it returned to normal at the time of presentation to our endocrine clinic. Oral contraceptives are associated with a two- to six-fold increased relative risk of developing venous thromboembolic events [15]. Atherosclerotic events such as stroke and myocardial infarction are also increased in those who use oral contraceptives [16,17]. However, to the best of our knowledge there have been no reports of patients on oral contraceptives developing a ventricular thrombus without myocardial infarction. In addition, the risk of thrombosis induced by oral contraceptives is highest in the first year of use [16,17] and the risk decreases with duration of use [18]. Therefore, oral contraceptives are less likely to be a major cause of our patient's ventricular and arterial thrombus, considering she has arterial but not venous thrombosis and thrombosis occurred after a number of years of oral contraceptive pill use. The association of hyperhomocysteinemia, a possible result of MTHFR mutation, with arterial vascular diseases or venous thrombosis is controversial [19-22]. The MTHFR defect, when combined with additional thrombophilic risk factors, is likely to increase the risk of venous thrombosis, especially for a patient with a homozygous mutation. The effect is uncertain when no additional risk factors are present and when the homocysteine level is only mildly elevated (< 30 umol/L) [21]. As far as we know, no studies have reported the association of a ventricular thrombus with MTHFR mutation. Furthermore, the mildly abnormal homocysteine level (14 umol/L) obtained on our patient was not a fasting value, therefore it is not very useful considering homocysteine level could be affected by dietary protein intake. Thus, the heterozygous mutation for MTHFR our patient has is unlikely to contribute significantly to her arterial and ventricular thrombosis. Although there is no direct proof that pheochromocytoma caused thrombosis in this patient, it probably contributed significantly to this process based on the aforementioned reasons. MTHFR heterozygous mutation and oral contraceptives may have contributed to this process but the likelihood is low. Appropriate anti-coagulation is essential for patients with pheochromocytoma and thrombosis to prevent devastating outcomes.

## Conclusion

We report a case of pheochromocytoma uniquely presenting with left ventricular thrombus and left axillary artery thrombus. This case highlights the complexity of managing patients with pheochromocytoma, and presents the possible association of pheochromocytoma with arterial thrombosis. Knowledge of this association and the potential for embolic events will educate clinicians to be more vigilant about the pro-thrombosis state in patients with pheochromocytoma. Anticoagulation regimen should be employed to avoid devastating embolic events and therefore reduce morbidity and mortality. This will help to make a difference in the management of patients with pheochromocytoma.

### Patient's perspective

The heart palpitations were the first symptoms I noticed. Those began in December 2001. By February 2002, I experienced my first migraine along with the palpitations. The migraine lasted several hours. I began taking oral contraceptives again in 2002 and the frequency and duration of the migraines diminished although not completely.

My symptoms were easy to ignore until the summer of 2005 when the "cluster" headaches started. These were different than migraines in that they started at the base of my skull on the right side and did not respond to over-the-counter pain relievers. Also during that time, I noticed my blood pressure was higher than normal. Historically, my blood pressure was in the low-to-normal range until about 2005, typically around 112/60. I had my blood pressure checked about once a year during routine physicals required to renew my birth control prescription. From 2005-2007, my blood pressure steadily rose. Beginning in 2006, I was treated with lisinopril and hydrochlorothiazide and later, atenolol. Once treatment began, the cluster headaches diminished.

Although the headaches diminished with the hypertension treatment, other symptoms became more apparent. Along with the "pounding" heart palpitations, I began experiencing a very definite progression of symptoms; pounding heart, a burning sensation around my heart and chest, cold sweats, nausea, vomiting. The burning and numbness would then creep up from my chest to the back of my neck and head and extend up. I would then experience excruciating headaches from the top of my head to behind my eyes. I called it "riding the wave" because they generally only lasted a few seconds to a few minutes and once the headache subsided, I felt fine. I could sometimes predict them when I noticed blind spots in my vision, the precursor to a migraine.

After the removal of the pheochromocytoma, the only negative health issues were an upper gastrointestinal bleed due to a Dieulafoy's Lesion, that occurred two weeks post op. Restenosis of my stent occurred in January 2008 which was discovered after my left arm went numb again. As a result of the blockage, my doctor has continued a prescription for Coumadin (warfarin) as well as 81 mg of aspirin. I also take one tablet of Folgard, a folic acid supplement. Other than those two incidents, I have felt fine and life is back to normal.

### Consent

Written informed consent was obtained from the patient for publication of this case report and any accompanying images. A copy of the written consent is available for review by the Editor-in-Chief of this journal.

## Competing interests

The authors declare that they have no competing interests.

## Authors' contributions

RH collected the data, took care of the patient and drafted the manuscript. AL performed the histological examination and edited the article. BTR wrote the patient's perspective. All authors read and approved the final manuscript.
